# Joint action on monitoring injuries in Europe (JAMIE)

**DOI:** 10.1186/0778-7367-70-19

**Published:** 2012-08-28

**Authors:** W H J Rogmans

**Affiliations:** 1 European Association for Injury Prevention and Safety Promotion (EuroSafe), Amsterdam, The Netherlands

## Abstract

**Background:**

Injuries due to accidents or violence constitute a major public health problem globally and also within the 27 member states of the European Union (EU-MSs). In spite of the magnitude and the severity of the problem, injury surveillance systems are not yet sufficiently well developed to accurately quantify the burden of injuries on individuals, health services and society in the EU-region. Much of the injury information generated up until now is not comparable between countries, and not between registers, due to the lack of harmonised methodology and classification.

The hospital sector provides the best setting for collecting information as this information relates to the most severe cases (while less severe cases are treated by family doctors of school nurses for instance) and information can be obtained easily on a large number of cases at low cost (while surveys are expensive and suffering serious deficiencies as regards the specificity of data obtained). The WHO-International Classification of Diseases and its derivative classification on external causes of injuries provide the proper tools for standardised data collection on injuries treated within the health sector.

**Project Objectives:**

JAMIE project aims at having by 2015 a common emergency departmental-based surveillance system for injury prevention in operation in all MS. Such a system should report on external causes of injuries due to accidents and violence as part of the Community Statistics on Public Health. The project will build on previous work on injury data exchange initiated by the European Commission (EC) and a number of EU-member states, which resulted to the so called Injury Data Base hosted by the EC.

In order to make injury data collection affordable for countries to collect and to have a greater number of countries joining the data exchange efforts, JAMIE envisages to have a relatively limited set data elements being collected in a representative sample of emergency departments in countries, while collecting in a few departments deeper information on the circumstances of the injury event.

## Background

Injuries due to accidents or violence constitute a major public health problem globally and also within the 27 member states of the European Union (EU-MSs). Within the EU-region, each year injuries result in an estimated 256,000 deaths, 7,200,000 hospital admissions, a further 34,800,000 emergency department (ED) attendances and 18,600,000 other medical treatments, totalling 60,600,000 medical treatments [1].

Injuries are commonly defined as being "caused by acute exposure to physical agents such as mechanical energy, heat, electricity, chemicals, and ionizing radiation interacting with the body in amounts or rates that exceed the threshold of human tolerance. In some cases (e.g. drowning and frostbites) injuries result from sudden lack of essential agents such as oxygen or heat" [2].

In spite of the magnitude and the severity of the problem, injury surveillance systems are not yet sufficiently well developed to accurately quantify the burden of injuries on individuals, health services and society in the EU-region.

What information is available tends to focus on fatal injuries. So also most of the targets of EU- and national policies with respect to road traffic safety, safety at work, consumer safety, violence and suicide prevention have been primarily focused on the reduction of deaths. However, deaths are only one aspect of the total injury problem; for every person killed, many more are seriously and permanently disabled and many more again suffer minor, short-term disabilities. Not only the costs of injury mortality but also the costs of morbidity are immense, not only in terms of lost economic opportunity and demands on national health budgets, but also in terms of personal suffering.

It is now increasingly acknowledged that deaths are only one measure of the magnitude of the injury problem. In fact, in many EU Member States deaths in road traffic or for instance at work, have been declining over the last several decades due in part to improvements in medical care (prompt emergency response, early diagnosis, and treatment capabilities) as well as to advances in road and vehicle design and in technology. In contrast to this development, non-fatal injuries are increasing in importance in terms of both societal and economic costs as well as loss of productivity. Consequently, there is a growing need for separate targets related to the reduction of non-fatal injuries, in particular those leading to permanent impairments. Such indicators are gradually being introduced at the EU level for target setting and for measuring progress in policies for road safety and for health and safety at work.

Much of the injury information generated up until now is not comparable between countries, and not between registers, due to the lack of harmonised methodology and classification. Injury surveillance in the EU – and in most MSs – can be characterized as operating on an incomplete puzzle of data sources that only provides a notion of the complete picture but lacks important details [3]. However these challenges can be met by using health based data that provide the ‘cement’ to glue the jigsaw pieces of understanding the injury field together and will serve as common denominator for all policy sectors and MSs.

It is obvious that the hospital sector provides the best setting for collecting information as this information relates to the most severe cases (while less severe cases are treated by family doctors of school nurses for instance) and information can be obtained easily on a large number of cases at low cost (while surveys are expensive and suffering serious deficiencies as regards the specificity of data obtained).

Technological developments in medical administration and data linkage, also offers new opportunities for recording information that is also relevant for injury prevention.

### EU-policy response

In response to the growing but incomplete evidence on the scale of the injury problem the EU Council issued in 2007 a Recommendation on the Prevention of Injuries and the Promotion of Safety [4] that urged all member states to develop national injury surveillance and reporting systems. The Council also invited the Commission to establish a Community-wide injury surveillance system to make the information contained in the database easily accessible to all stakeholders.

Over the past few years, several projects have been initiated by the Commission to develop such an exchange of injury data at the EU level based on data collected in accident and emergency departments at general hospitals. In 2010, thirteen EU-MSs were routinely collecting injury data in a sample of hospitals and delivering these data to the Commission, in line with the Injury Data Base (IDB) methodology [5]. This methodology allowed countries to collect accident and injury data from a representative sample of emergency departments in the participating countries and to use a standardised classification for coding the circumstances of the injury-event and its outcome (as a derivative classification of the WHO-International Classification of External Causes of Injuries, ICECI [6]).

IDB-system complements existing data sources such as the routine causes of death statistics, hospital discharge registers and data sources specific to injury areas, including road accidents and work related accidents. Currently 13 countries are still collecting injury data in line with this methodology, although some only for a selected population, e.g. by collecting information in pediatric hospitals only or by collecting only injuries due to home and leisure accidents.

### A new three year injury-data collection initiative

In order to encourage continuation of these data collection efforts and the inclusion of the remaining EU-MSs in EU-wide injury data exchange, the European Commission, DG for Health and Consumers (DG Sanco) is currently funding a public health Joint Action on injuries known as JAMIE (Joint Action on Monitoring Injuries in Europe) from 2011–2013. The project is being endorsed by the Ministries of Health in 22 EU-MSs (*see list in*Appendix I). Each of these Ministries have designated a internal unit or a national competent organisation to contribute to the JAMIE-project and to test the feasibility of introducing an pragmatic and sustainable injury surveillance in their country.

The JAMIE project aims at having by 2015 a common hospital-based surveillance system for injury prevention in operation in all MS. Such a system should report on external causes of injuries due to accidents and violence as part of the Community Statistics on Public Health.

Up to mid 2014 a series of actions are envisioned that will lay the groundwork for a genuine EU-wide injury information system through the following steps:

within twelve months criteria for IDB data quality, such as representativeness and comparability (taking into account the differences in the organisation of emergency services in countries), will be clearly defined, in line with the respective requirements of the European Statistical System (ESS); and

over the years 2012–2014 an increasing number of countries will be assisted in collecting and injury data in accordance with these quality criteria for uploading in the EU central Injury Database (IDB), hosted by the European Commission, DG Health and Consumers.

By the end of the action (mid 2014), in at least 26 countries National IDB Data Administration Centre (’NDA’) shall be designated by the competent national or regional authority and be in full operation, and at least 22 countries shall collect IDB data in a sustainable manner. Four more countries are expected to have implementation plans in place endorsed by the competent authorities.

### The JAMIE-approach

Whilst from an EU perspective the main focus of JAMIE is to develop a system to enable the incidence of home and leisure injuries to be monitored by an increasing number of countries, it is clear that there are many other needs for injury data to support policy development, appraisal, prevention and research in relation to injuries from defective products, or resulting from violence or road traffic accidents, to name but a few. With not too much effort and within the existing resources provided through JAMIE it would be possible to provide tools to answers most of these questions at individual member state or EU level. Big samples of MDS are needed for accurate estimates for incidences (not only for home and leisure injuries). Additional in-depths information on external causes, circumstances, locations, activities, and products are needed for developing preventive measures, guiding and evaluating prevention programmes.

That is why JAMIE allows EU-MSs to supply ED injury data with two levels of depth on injury determinants, the minimum and full level datasets. The combination of much larger amounts of cases at a lower level of detail as to the injury circumstances, sufficient for developing the accurate estimates of population incidence, with data at high levels of detail from a relatively small number of hospitals provides information for a wide range of policy makers and health, transportation and consumer protection authorities.

The proposed two level system involves the implementation of emergency department datasets at different levels of sophistication: 1. the Full injury surveillance Data Set (FDS, previously implemented as the Injury Data Base or IDB); and 2. a new Minimum Data Set (MDS).

Decision about the content of these datasets has been based on a review of the existing literature and practices around the world and discussion between experts on the feasibility of collecting such data whilst ensuring consistency as far as possible with existing classification systems. The proposed MDS-Injury is now presented in Table 1 as a single screen reporting tool. This data set serves as complementary items to the data elements related to the 'nature of injury' and 'body part affected' that are already collected in all emergency departments in a routinely manner.

**Table 1:** The single screen minimum data set: external factors of injury

**Intent:**	**Selected mechanisms:**
–Accidental injury	–Fall
–Deliberate (intentional) self harm	–Cut/pierce
–Assault related injury	–Road traffic
–Unknown intent	–Poisoning
	–Burn/scald
	–Other
	–Unknown
**Location (setting):**	**Selected activities:**
–Workplace	–Sport
–Road (incl. pavement)	–Work
–Educational establishment	–Other
–Leisure area (incl. sport/fitness, shops, pubs, clubs and recreation)	–Unknown
–Home (includes garden)	
–Other (includes health facilities)	
–Unknown	

The MDS is designed to be implemented in many different ways, including the creation of de novo computer systems, the adaptation of existing systems, or using check boxes in existing or new paper based clinical records.

The simple MDS for Europe reflects the need to meet many different agendas in relation to data collection, such as supporting the development of high level EU and MS injury indicators, being feasible to implement in MSs with wide variation in existing practice, and maximising the potential to support prevention and research.

The proposed Full Data Set (FDS) is in line with the original IDB-classification as it has been implemented over the past few years in 13 countries [1]. The categories of external cause variables included in the proposed FDS (see Appendix II) reflect the responsibility of the major agencies and bodies involved in prevention in many countries, including the prevention of injuries in specific domains such as road traffic, consumer products and services, or in work environments. In creating such a dataset we have been guided by the need to be able to capture the required variables efficiently and from a variety of staff in emergency departments including reception staff and clinicians. In response to the latter requirement we have chosen terminology for categories which are widely understood both by the general public and clinical staff, e.g. put ‘accidental injury’ instead of ‘unintentional injury’ on the form.

### Health policy use

Given the range of data being collected as part of the MDS proposed in the JAMIE project, including information on the age/gender of the injured individual, the nature of the injury sustained, the mechanism of the injury and the activity/location/intent associated with the injury, the opportunity exists for each member state to use this information to calculate the number of DALYs and the size of the direct medical costs applicable to their own country. Such information is extremely valuable for undertaking economic analyses to assess the effectiveness or cost-benefit of injury prevention strategies in the EU-region.

Due to the complexities of these calculations the project will provide instructions relating to how DALYs and direct medical costs can be measured, utilising the knowledge gained and findings resulting from the GBDI study [7] and the UK Burden of Injury (UK BOI) study [8].

The complexity of the project as well as the diversity of stakeholders involved calls for a comprehensive communication plan in order to ensure focus in all activities and among all involved partners. The project group will work closely together with the competent authorities in MSs and those involved in the development of health sector based injury data exchange, including the European Statistical Office and World Health Organization ICD Revision Group.

## Appendix I

### Who we are

To date, the following countries joined the project as associated partners in JAMIE:

**Table:** 

	
Austria, Kuratorium für Verkehrssicherheit	Lithuania, Institute of Hygiene
Cyprus, Ministry of Health	Latvia, National Health Services
Czech Republic, The University Hospital Brno	Malta, Directorate General Strategy & Sustainability
Denmark, Syddansk Universitet	Netherlands, Consumer Safety Institute
Estonia, Ministry of Social Affairs of Estonia	Norway, Norwegian Safety Forum
Germany, Landesgesundheitsamt Brandenburg	Portugal, Instituto Nacional de Saúde-Dr. Ricardo Jorge IP
Greece, National School of Public Health	Romania, Babeş-Bolyai University Cluj-Napoca
Hungary, National Institute for Health Development	Sweden, National Board of Health and Welfare
Ireland, National Suicide Research Foundation	Slovenia, National Institute of Public Health
Iceland, The Directorate of Health	Spain, Servicio Navarra de Salud
Italy, Instituto Superiore di Sanità	United Kingdom, Swansea University

Collaboration has been established with a number of other countries, including Luxembourg, Poland and Croatia.

The JAMIE-project has been initiated, with the endorsement of governments in the EU-Member States, by a consortium of centres of excellence in injury surveillance based in the EU region:

· the Austrian Road Safety Board (KfV), Vienna, Austria;

· the European Association for Injury Prevention and Safety Promotion (EuroSafe), Amsterdam, the Netherlands;

· the National Institute for Health Development (NIHD), Budapest, Hungary;

· the Swansea University School of Medicine, Health Information Research Unit (SU), Swansea, Wales, UK; and

· the Brandenburg University of Technology, Information Systems Unit, Cottbus, Brandenburg, Germany.

The European Association for Injury Prevention and Safety Promotion (EuroSafe) provides leadership to the project.

## Appendix II

### Core IDB FDS data elements

Recording country – Country that provides the dataUnique national record number – Number of the emergency department case or recordAge of patient – Person’s age at the time of the injurySex of patient – Person’s sex at the time of the injuryCountry of permanent residence – Person’s country of residence at the time of the injuryDate of injury – The date the injury was sustainedTime of injury – The time the injury was sustainedDate of attendance – The date the injured person attended the Emergency DepartmentTime of attendance – The time the injured person attended the Emergency DepartmentTreatment and follow-up – Status of treatment after attendance at the Emergency DepartmentIntent – The role of human purpose in the injury eventTransport injury event – Any incident involving a transport device and resulting in injuryPlace of occurrence – Where the injured person was when the injury event startedMechanism of injury – The way in which the injury was sustained (i.e. how the person was hurt)Activity when injured – The type of activity the injured person was engaged in when the injury occurredObject/substance producing injury – Matter, material or thing being involved in the injury eventType of injury – Type of injury sustainedPart of the body injured – Region or part of the body where the injury is locatedNarrative – Description of the event leading to the (suspected) injury

### Additional IDB data elements

#### Admission module

Number of days in hospital – The number of days the injured person is admitted in the recording hospital.

#### Violence module

Victim/perpetrator relationship – The relationship of the person committing the violent act to the injured person.Sex of perpetrator – The sex of the person who inflicted the injury.Age group of perpetrator – The age group of the person who inflicted the injury.Context of assault – The circumstances surrounding the violent injury event.

#### Intentional Self-harm module

Proximal risk factor – The most recent crises that led to the self-harm incident.Previous intentional self-harm – Whether or not the injured person attempted intentional self-harm before.

#### Transport module

Mode of transport – The means by which the injured person was travelling from one place to another.Role of the injured person – How the injured person was involved with the specified mode of transport at the time of the injury event.Counterpart – The other vehicle, object, person, or animal (if any) with which the injured person, or the vehicle in which the injured person was travelling, collided.

#### Sports module

Type of sport/exercise activity – The type of sport or exercise activity in which the injured person was engaged at the time of the injury.
